# A Real-Life Study of Prolonged Meropenem Infusion in Neonates and Children Admitted to Intensive Care Units: Are Three Hours Long Enough?

**DOI:** 10.3390/jcm14051488

**Published:** 2025-02-23

**Authors:** Marcello Mariani, Marco Scaglione, Chiara Russo, Andrea Rainelli, Alessio Mesini, Carolina Saffioti, Erica Ricci, Alessia Cafaro, Giuliana Cangemi, Martina Bavastro, Tommaso Bellini, Giacomo Brisca, Andrea Moscatelli, Elio Castagnola

**Affiliations:** 1 Pediatric Infectious Diseases Unit, Department of Pediatrics, IRCCS Istituto Giannina Gaslini, 16147 Genoa, Italy; 2 Department of Neuroscience, Rehabilitation, Ophthalmology, Genetics, Maternal and Child Health (DINOGMI), University of Genoa, 16132 Genoa, Italy; 3 Unit of Biochemistry, Pharmacology and Newborn Screening, Central Laboratory of Analysis, IRCCS Istituto Giannina Gaslini, 16147 Genoa, Italy; 4 Division of Infectious Diseases, Department of Health Sciences (DISSAL), University of Genoa, 16132 Genoa, Italy; 5 Pediatric Emergency Room and Emergency Medicine Unit, Emergency Department, IRCCS Istituto Giannina Gaslini, 16147 Genoa, Italy; 6 Neonatal and Pediatric Intensive Care Unit and Intermediate Care Unit, Emergency Department, IRCCS Istituto Giannina Gaslini, 16147 Genoa, Italy

**Keywords:** meropenem, critically ill, children

## Abstract

**Background/Objectives**: Meropenem is a broad-spectrum antibiotic essential for treating resistant Gram-negative infections in pediatric patients. Current dosing recommendations may not consistently achieve optimal pharmacokinetic (PK) targets, especially in critically ill children. **Methods**: We conducted a retrospective cohort study at IRCCS Istituto Giannina Gaslini, analyzing 97 plasma levels from 86 pediatric patients (<18 years) hospitalized between January 2020 and December 2023 in the neonatal and pediatric intensive care unit. Patients receiving meropenem for proven or suspected infections were included. Demographic, clinical, and PK parameters were assessed, with a focus on trough concentrations (C_trough_). **Results**: The median age was 25 months, with neonates representing 15.5% of cases. The median C_trough_ was 2.8 mg/L and was significantly higher in neonates (8.9 mg/L) compared to older patients (2.2 mg/L, *p* < 0.001). Only 27.8% of patients achieved the target C_trough_ of >8 mg/L, with estimated glomerular filtration rate (eGFR) being the primary factor influencing these levels. Patients with C_trough_ > 8 mg/L had a significantly lower eGFR (61 mL/min/1.73 m^2^) compared to those below this threshold (131 mL/min/1.73 m^2^, *p* = 0.001). **Conclusions**: The current meropenem dosing regimen may not reliably meet PK targets in critically ill pediatric patients, particularly those with augmented renal clearance or when treating pathogens with increased meropenem MIC. Our findings suggest that increased dosages and prolonged infusion times may be necessary to optimize therapeutic efficacy against resistant Gram-negative bacteria in this vulnerable population. Further studies are needed to refine dosing strategies and improve patient outcomes.

## 1. Introduction

Meropenem is an antibiotic belonging to the carbapenem family, a class of broad-spectrum molecules with a time-dependent mechanism of action that are effective against most Gram-negative bacteria [1]. Meropenem is also effective against multidrug-resistant pathogens, including extended-spectrum β-lactamase (ESBL)-producing and ampicillinase C (AmpC)-producing *Enterobacterales*, *Pseudomonas aeruginosa* and *Acinetobacter baumannii*, which pose significant challenges in the treatment of nosocomial infections [2,3]. It is administered intravenously, and its pharmacokinetics (PK) allow for rapid distribution in tissues and good penetration into body fluids, such as cerebrospinal fluid, making it a viable option for treating bacterial meningitis [4]. To achieve optimal efficacy, the free drug plasma concentration must be above the pathogen minimum inhibitory concentration (MIC) for at least 40% of the time between dosing intervals (40% *f*T > MIC), but in severe infections, higher percentages (70% to 100% *f*T > MIC) could be required [5]. In recent years, several studies have focused on the pharmacokinetics of meropenem, leading to some concerns about treatment protocols also used in pediatric patients. For pediatric patients with normal renal function, the dosage of meropenem approved by major drug regulatory authorities such as the European Medicines Agency (EMA) and the United States Food and Drug Administration (FDA) ranges from 10 to 40 mg/kg every 8 h, administered in 15–30 min or via bolus injection (5–20 mL) in 3–5 min. The maximum recommended dosage of 40 mg/kg every 8 h applies specifically to bacterial meningitis in children aged 3 months and older [6,7]. Off-label dosing through prolonged infusion (at least over 2–3 h) has been found to be superior to current the FDA- and EMA-recommended prescription, particularly for infections caused by resistant bacteria [1,8,9].

Gram-negative bacteria can acquire carbapenem resistance both through the production of hydrolyzing enzymes (carbapenemases) and through non-enzymatic mechanisms (e.g., mutation of porin genes) [10]. Maintaining high plasma levels of meropenem for an extended duration has proven beneficial in overcoming elevated minimum inhibitory concentrations (MICs), such as 8 or 16 mg/L [11].

Although conflicting data emerged from an adult randomized controlled trial assessing the efficacy of 24 h continuous infusion in reducing mortality [12], in pediatric patients, prolonged meropenem infusion has been associated with better clinical outcomes and lower mortality and has been shown to be the only effective schedule to achieve pharmacokinetic/pharmacodynamic (PK/PD) objectives in selected categories, such as children on extra-corporeal membrane oxygenation (ECMO) and continuous renal replacement therapy (CRRT) [13,14,15], solid organ transplant recipients [16] and critically ill patients [17]. In addition, recent data suggest that continuous infusion over 24 h is the preferred dosing regimen for infections caused by pathogens with higher MICs in children [18].

Due to concerns about the efficacy and effectiveness of currently registered meropenem dosing, as well as the relatively limited availability of pediatric cohorts, the aim of our work is to evaluate the achievement of PK targets in critically ill pediatric patients treated with prolonged meropenem infusion.

## 2. Materials and Methods

We conducted a retrospective single-center cohort study on pediatric patients (<18 years) hospitalized at IRCCS Istituto Giannina Gaslini, a large third-level pediatric hospital, in Genoa (Italy) from 1 January 2020 to 31 December 2023.

Patients were included if they were hospitalized in the pediatric or neonatal intensive care unit and received meropenem at 20–40 mg/kg over a prolonged infusion of 2/3 h every 8 h, with pre-dose plasma levels (C_trough_) evaluated before at least the 5th dose. The PK target was considered as C_trough_ > 8 mg/L, the meropenem breakpoint MIC for *Enterobacterales* [19].

If more than one plasma level was available, only the first one was considered within the same treatment cycle. Meropenem levels are routinely monitored as part of standard care at our center, with a target C_trough_ of ~8 mg/L. Subsequent levels were excluded, as they may reflect dose adjustments to achieve this target. A further level was considered in the same patient only if a new treatment was started after at least 4 weeks. Patients were excluded if on meropenem treatment without any plasma level assessment, and if on CRRT or ECMO support.

Meropenem plasma levels were assessed by a previously published liquid chromatography-tandem mass spectrometry (LC-MS/MS) method [20]. Total drug concentration was assessed but, since meropenem protein binding is only about 2% [6], this is representative of the free (and pharmacologically active) fraction.

Demographic, microbiological and laboratory data, as well as pharmacokinetic (PK) parameters (infusion time, administered mg/day, meropenem plasma levels), were anonymously collected. For each patient, the glomerular filtration rate (eGFR) was estimated by means of the modified Schwartz formula [21].

Mean and standard deviation (SD) were presented for normally distributed variables while median and interquartile ranges (IQR) were used for non-normally distributed variables. Numbers and percentages were utilized to represent categorical variables. Parametric tests (*t*-test) were used to compare groups for continuous variables with normal distribution, while non-parametric tests (Mann–Whitney) were used for non-normally distributed variables. For categorical variables, Pearson’s χ^2^-test or Fisher’s exact test were performed when appropriate. The association between meropenem C_trough_ levels and potential explanatory variables was assessed by means of multivariable binomial regression in which only variables found to be significant in univariate analysis were included. Collinearity among variables was assessed using the variance inflation factor (VIF) before inclusion in the multivariable model to avoid distortions. The analysis was performed using Jamovi software Version 2.6.23 (Jamovi Project, 2021, https://www.jamovi.org (Accessed on 30 January 2025)).

## 3. Results

A total of 97 plasma levels were obtained from 86 patients, comprising 36 females (41.9%) and 50 males (58.1%). The median age at the sample time was 25 months (2 years), with an IQR of 145 months (12 years). Of the patients, 15.5% were younger than 1 month (neonates), with a median gestational age of 39 weeks (IQR 10). Table 1 summarizes differences in clinical variables between neonates and older patients.

Overall, the median meropenem C_trough_ was 2.8 mg/L, with significantly higher levels in neonates (8.9 mg/L) than in older patients (2.2 mg/L) (*p* < 0.001) at a median real administered dose of 60 mg/kg/day and a median infusion time of 3 h/dose for both age groups.

Other variables with statistically significant differences between neonatal and pediatric populations include median plasma albumin (3161 mg/dL versus 3497 mg/dL), median alanine aminotransferase (ALT) (11 U/L versus 26 U/L), and median total bilirubin (5 mg/dL versus 0.51 mg/dL).

The proportion of patients with C_trough_ > 8 mg/L was 27.8% (27/97); among them, 8/27 (29.6%) were neonates. Median eGFR was significantly lower in patients with C_trough_ plasma concentrations above 8 mg/L (61 vs. 131 mL/min/1.73 m^2^; *p* = 0.001); eGFR was the only statistically significant variable in the multivariable analysis and was lower in neonates (33 mL/min/1.73 m^2^) than in other patients (131 mL/min/1.73 m^2^, *p* < 0.001), as reported in Table 2.

**Figure 1:**
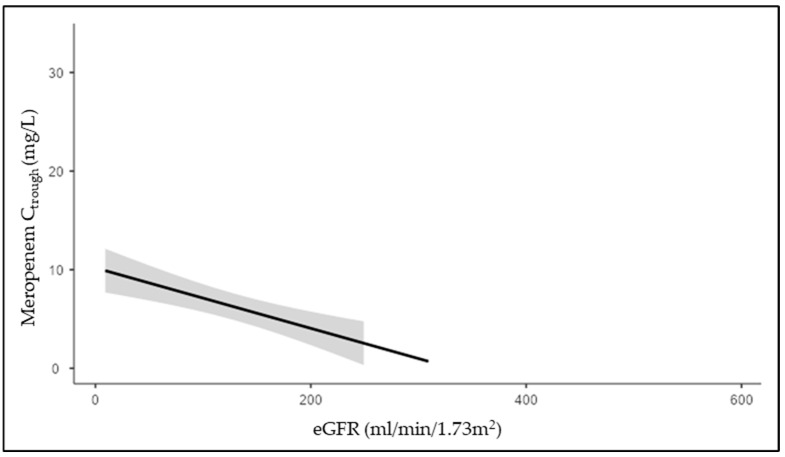
Meropenem C_trough_ and eGFR inverse correlation.

A statistically significant inverse correlation was found between eGFR and meropenem C_trough_ (r_s_ −0.582; *p* < 0.001), as shown in Figure 1. Based on our data, in our population with an estimated GFR of 125 mL/min/1.73 m^2^, the estimated median meropenem C_trough_ is 5.9 mg/L (95% confidence interval 4.74–7.25).

Pathogens were identified in 60/97 (61.9%) episodes, almost exclusively from blood cultures. The most frequent was *Pseudomonas aeruginosa* (17/60, 28.3%), followed by *Klebsiella pneumoniae* (12/60, 20%), *Escherichia coli* (9/60, 15%), *Serratia marcescens* (5/60, 8.3%), *Klebsiella oxytoca* (4/60, 6.7%), *Enterobacter cloacae complex* (2/60, 3.3%), *Proteus mirabilis* (2/60, 3.3%) and other fermentative Gram-negative bacilli. The median meropenem MIC of bacterial isolates was 0.12 mg/L.

## 4. Discussion

The present study reports data from a real-life pediatric and neonatal cohort in an intensive care setting. According to our results, in less than 30% of cases, prolonged meropenem infusion in critically ill patients lead to a 100% *f*T > breakpoint MIC for *Enterobacterales*.

A significant difference in median meropenem C_trough_ was observed between neonates and older patients (with the former exhibiting median levels approximately eightfold higher). However, the only independent parameter for achieving C_trough_ > 8 mg/L was found to be eGFR.

This strict dependence is likely attributable to the molecular characteristics of meropenem, which exhibits a 2% protein binding rate and predominantly undergoes renal excretion [22]. Patients who are admitted to the ICU are susceptible to developing a condition known as “augmented renal clearance” (ARC), which is characterized by an increase in renal function resulting from an inflammatory response, fluid therapy, inotropes, or other intensive care procedures. Depending on the authors, ARC is defined by an eGFR between 120 and 160 mL/min/1.73 m^2^, although the most commonly reported threshold is eGFR > 130 mL/min/1.73 m^2^ [23]. In patients with ARC, the proportion of free drug excreted by urinary route increases, consequently reducing plasma levels and negatively impacting PTA. Furthermore, glomerular filtration is a dynamic process that increases progressively from birth to weeks after delivery and is due to changes in blood flow and renal vascular resistance. Gestational age correlates with the eGFR at birth, which is further reduced in preterm infants [24,25].

In the neonatal cohort under investigation, the median eGFR is 33 mL/min/1.73 m^2^. This reduced renal clearance has a positive influence on median meropenem levels, whose C_trough_ is significantly higher than in the non-neonatal cohort. Conversely, median eGFR of non-neonatal patients in our cohort exceeded the ARC threshold of 130 mL/min/1.73 m^2^, favoring the increased urinary elimination of meropenem and resulting in significantly lower median C_trough_ plasma concentrations of approximately 2 mg/L.

As ARC has been found to have a prevalence as high as 78% among children [26], it is essential to routinely evaluate renal function. Regular assessment is critical not only for identifying renal insufficiency, which necessitates adjustments to drug dosages to avoid toxicity, but also for diagnosing ARC. In this condition, higher drug dosages or the prolongation of infusions may be required, potentially extending to continuous administration, particularly for antibiotics with low protein binding.

Our study has several limitations. First, it was a retrospective, observational analysis in which only a single plasma level of meropenem per infectious episode was assessed without evaluating the pharmacokinetics of the drug in the subsequent days of treatment. Second, although an ideal PK of 100% a *f*T > breakpoint was not reached in most cases, the standard dosing regimen of meropenem would likely have preserved its efficacy. This conclusion is supported by the median minimum inhibitory concentration (MIC) of 0.12 mg/L for the pathogens isolated in our cohort, which is well below the established breakpoint threshold due to the low incidence of carbapenem-resistant pathogens in our center. When considering the actual MICs instead of a hypothetical 8 mg/L, target achievement rates were notably high: in 78.3% of cases, the C_trough_/MIC ratio exceeded 4; however, in 10% of cases, the C_trough_/MIC ratio was below 1, raising concerns about potential suboptimal efficacy in a non-negligible proportion of patients. This underscores the importance of individualized therapy and the role of therapeutic drug monitoring in optimizing outcomes, even in populations with favorable MIC distributions.

For this reason, a very stringent PK parameter (C_trough_ above the *Enterobacterales* breakpoint 100% of the time, although maintaining a *f*T > MIC of 60% is usually considered acceptable) was deliberately chosen given the escalating issue of antibiotic resistance in Gram-negative pathogens within critical care settings. This approach aims to minimize the selection of resistant mutants and enhance clinical outcomes.

Finally, the modified Schwartz formula, developed from a cohort of patients with renal disease, has been shown to yield lower values in pediatric patients with normal or high renal function compared to the “classical” 24 h measured urinary creatinine clearance (ClCr 24 h), potentially underestimating the prevalence of ARC [27]. It is also important to note that the Schwartz formula is not ideal for estimating the glomerular filtration rate (GFR) in neonates, as it may not accurately reflect renal function in this young population. Despite these limitations, the modified Schwartz formula remains the most accessible and practical tool for routine clinical use. It has been demonstrated not to be inferior to more complex equations in estimating ARC, thus serving as a valuable resource for clinicians seeking an uncomplicated and effective method for renal function assessment [28].

## 5. Conclusions

In our cohort, meropenem levels were primarily influenced by renal function, with no significant impact from age or other laboratory parameters. In critically ill patients, particularly those with augmented renal clearance (ARC), the standard pediatric dosing regimen for sepsis, even with prolonged infusion, may fail to meet the pharmacokinetic (PK) target of 100% *f*T > MIC for pathogens with elevated MICs.

Therefore, in critically ill pediatric patients with severe infections or resistant pathogens, increasing dosages and extending the infusion to 24 h may be necessary to ensure optimal therapeutic efficacy and treatment outcomes.

## Figures and Tables

**Table 1:** Laboratory tests differences among neonatal and non-neonatal cohorts. AST, aspartate aminotransferase; ALT, alanine aminotransferase; Gamma-GT, gamma glutamyl transferase; MIC, minimal inhibitory concentration.

Median (IQR)	Neonates N = 15	Non-Neonates N = 82	*p*
Meropenem C_trough_ (mg/L)	8.9 (6.2–12.5)	2.2 (0.925–7.35)	<0.001
Total plasmatic proteins (g/dL)	4.59 (4.1–5.6)	6.09 (5.8–6.9)	<0.001
Albumin (mg/dL)	3161 (2447–3575)	3497 (3154–3997)	0.05
AST (U/L)	38 (26–49)	31 (20–59)	0.693
ALT (U/L)	11 (6–23)	26 (17–53)	0.001
Gamma-GT (U/L)	41 (31–84)	71 (24–183)	0.345
Total bilirubin (mg/dL)	5.00 (1.9–8.5)	0.51 (0.2–1.2)	<0.001
eGFR (mL/min/1.73 m^2^)	33 (21.5–54.5)	131 (91–178)	<0.001
MIC (mg/L) of isolates	0.120 (0.08–0.12)	0.120 (0.05–0.5)	0.358
Administered dose mg/kg	60.0 (60–120)	60.0 (60–100)	0.970

**Table 2:** Meropenem C_trough_ differences. AST, aspartate aminotransferase; ALT, alanine aminotransferase; Gamma-GT, gamma glutamyl transferase; MIC, minimal inhibitory concentration.

Median (IQR)	Meropenem C_trough_ > 8 mg/L N = 27	Meropenem C_trough_ < 8 mg/L N = 70	*p* (Univariate)	*p* (Multivariable)
Meropenem C_trough_ (mg/L)	15.7 (10.3–19.4)	1.7 (0.7–3.4)		
Total plasmatic proteins (g/dL)	5.82 (5.5–6.3)	6.04 (5.2–6.9)	0.310	
Albumin (mg/dL)	3405 (3045–3805)	3473 (3113–3997)	0.419	
AST (U/L)	35 (25–64)	30 (20–53)	0.421	
ALT (U/L)	30 (13–49)	23 (14–51)	0.953	
Gamma-GT (U/L)	73 (27–173)	65 (22–127)	0.696	
Total bilirubin (mg/dL)	1.4 (0.6–2.6)	0.46 (0.2–1.2)	0.003	0.536
eGFR (mL/min/1.73 m^2^)	61 (28.5–123.5)	131 (87–175.5)	0.001	0.004
MIC (mg/L) of isolates	0.12 (0.05–0.375)	0.12 (0.12–0.5)	0.197	
Administered dose mg/kg	60 (60–100)	60 (60–100)	0.925	
Neonates (n, %)	8 (10)	7 (29.6)	0.027	0.300

## Data Availability

Data are available upon request from the corresponding author.
